# COVID-19 patient satisfaction and associated factors in telemedicine and hybrid system

**DOI:** 10.3389/fpubh.2024.1384078

**Published:** 2024-04-05

**Authors:** Dagmawit G. Gashaw, Zewdie Aderaw Alemu, Freddy Constanzo, Feben T. Belay, Yakob W. Tadesse, Carla Muñoz, Juan Pablo Rojas, Cristobal Alvarado-Livacic

**Affiliations:** ^1^National Public Health Emergency Operation Center, Ethiopian Public Health Institute, Addis Ababa, Ethiopia; ^2^Saint Paul’s Hospital, Milennium Medical College, Addis Ababa, Ethiopia; ^3^Neurology Unit, Hospital Las Higueras, Talcahuano, Chile; ^4^Medical Program in Adult Neurology, School of Medicine, Universidad Católica de la Santísima Concepción, Concepción, Chile; ^5^National Training Center, Ethiopian Public Health Institute, Addis Ababa, Ethiopia; ^6^Ministry of Health of Ethiopia, Addis Ababa, Ethiopia

**Keywords:** patient satisfaction, COVID-19, home care, telehealth, telemedicine, digital health

## Abstract

**Background:**

The quality assessment of the home-based isolation and care program (HBIC) relies heavily on patient satisfaction and length of stay. COVID-19 patients who were isolated and received HBIC were monitored through telephone consultations (TC), in-person TC visits, and a self-reporting application. By evaluating patient satisfaction and length of stay in HBIC, healthcare providers could gauge the effectiveness and efficiency of the HBIC program.

**Methods:**

A cross-sectional study design enrolled 444 HBIC patients who answered a structured questionnaire. A binary logistic regression model assessed the association between independent variables and patient satisfaction. The length of stay in HBIC was analyzed using Cox regression analysis. The data collection started on April (1–30), 2022, in Addis Ababa, Ethiopia.

**Results:**

The median age was 34, and 247 (55.6%) were females. A greater proportion (313, 70.5%) of the participants had high satisfaction. Higher frequency of calls (>3 calls) (AOR = 2.827, 95% CI = 1.798, 4.443, *p* = 0.000) and those who were symptomatic (AOR = 2.001, 95% CI = 1.289, 3.106, *p* = 0.002) were found to be significant factors for high user satisfaction. Higher frequency of calls (>3 calls) (AHR = 0.537, 95% CI = 0.415, 0.696, *p* = 0.000) and more in-person visits (>1 visit) (AHR = 0.495, 95% CI = 0.322, 0.762, *p* = 0.001) had greater chances to reduce the length of stay in the COVID-19 HBIC.

**Conclusion:**

70.5% of the participants had high satisfaction with the system, and frequent phone call follow-ups on patients’ clinical status can significantly improve their satisfaction and length of recovery. An in-person visit is also an invaluable factor in a patient’s recovery.

## 1 Introduction

The global COVID-19 pandemic has had a significant impact on numerous countries, resulting in a staggering number of confirmed cases and deaths worldwide. Specifically, 774,771,942 confirmed cases and 7,035,337 deaths were reported globally. In Africa, approximately 9,576,309 confirmed cases and 175,500 deaths have been recorded, whereas Ethiopia alone has reported 501,157 confirmed cases and 7,574 deaths (1). Given the outbreak’s severity and the prevailing epidemiological situation, the Federal Ministry of Health of Ethiopia has taken swift action by developing, approving, and implementing a national guide on home-based isolation and care (HBIC). This guide is tailored for asymptomatic and mildly confirmed COVID-19 cases (2). Consequently, many patients have enrolled in the system, establishing a comprehensive telephone consultation (TC) service. Healthcare workers from health centers have been diligently monitoring the clinical condition of these patients through regular phone calls or in-person visits (2). The national or regional COVID-19 emergency centers are available for assistance to ensure that patients receive the necessary support and information. Patients facing challenges or requiring guidance on the system’s functioning, worsening symptoms, medication advice, or ambulance requests can reach these centers (2). This approach provides comprehensive care and support to individuals affected by COVID-19 in Ethiopia.

Several studies have emphasized the utilization of teleconsultations (TC) in Sub-Saharan Africa amid the COVID-19 outbreak. For instance, a study conducted at the Aga Khan Hospital in Dar es Salaam, Tanzania, revealed varying frequencies of TC calls over 3 months, with peaks observed at 7 weeks and lows at 13 weeks (3). In Uganda, the Ministry of Health rolled out TC services across multiple health centers simultaneously (4). Similarly, in Cameroon, many physicians resorted to TC consultations during the pandemic, often using WhatsApp applications (5). Despite the progress in telemedicine practices, assessing the quality of care provided through these methods is crucial. Quality indicators for telemedicine outcomes include mortality rates, average length of stay, and complication rates (6). The adoption of telemedicine has played a pivotal role in enhancing the quality of diagnosis and treatment in primary public hospitals (7). This is evident in the reduction of treatment duration, decrease in the average length of hospital stays, and decline in the percentage of critically ill patients (8). Moreover, telemedicine aids in improving personalized care and broadening the accessibility of medical services. Healthcare providers can deliver more efficient and effective patient care by harnessing telemedicine, particularly during crises such as the COVID-19 pandemic (9, 10). Patient satisfaction is a significant indicator of the societal perception of healthcare services, particularly in telemedicine. The practical advantages of telemedicine further underscore its social reputation within primary healthcare facilities. First, Telemedicine plays a crucial role in alleviating the financial burden on patients and their families by eliminating expenses associated with transportation and accommodation (11). Second, the efficient nature of telemedicine expedites disease diagnosis and treatment, thereby mitigating patients’ suffering (11). Lastly, telemedicine’s unique “face-to-face” communication system fosters a novel connection between medical professionals and patients, ultimately enhancing the healthcare experience (12, 13). Despite numerous studies, the findings remain inconclusive and conflicting. Some researchers suggested recruiting adaptable patients who embrace this new technology’s convenience (14). Conversely, others highlight limitations, such as the relative novelty of telehealth in medicine, which makes it challenging to compare its outcomes with those of more traditional interventions (15). In addition, patient satisfaction is a complex and multifaceted concept influenced by various factors. However, the factors influencing patient satisfaction in different settings or conditions remain debated (15).

Patient satisfaction and length of stay are crucial metrics for assessing the quality of healthcare programs. This study aimed to provide insights into the factors influencing the satisfaction and length of stay in HBIC of COVID-19 patients in Addis Ababa, Ethiopia. This study uses traditional care (TC) and hybrid (in-person-TC) approaches in different sub-city areas. By understanding these factors, policymakers can develop strategic plans to improve the quality of healthcare services, thereby ensuring high satisfaction levels and shorter medical residence stays. The findings of this study can also inform the National Public Health Emergency Operation Center and the Federal Ministry of Health in Ethiopia on how to intervene and strengthen the healthcare system. In addition, the results can contribute to expanding telemedicine services and adopting digital health technologies in the country’s healthcare sector.

## 2 Methods

### 2.1 Study area

**Figure 1:**
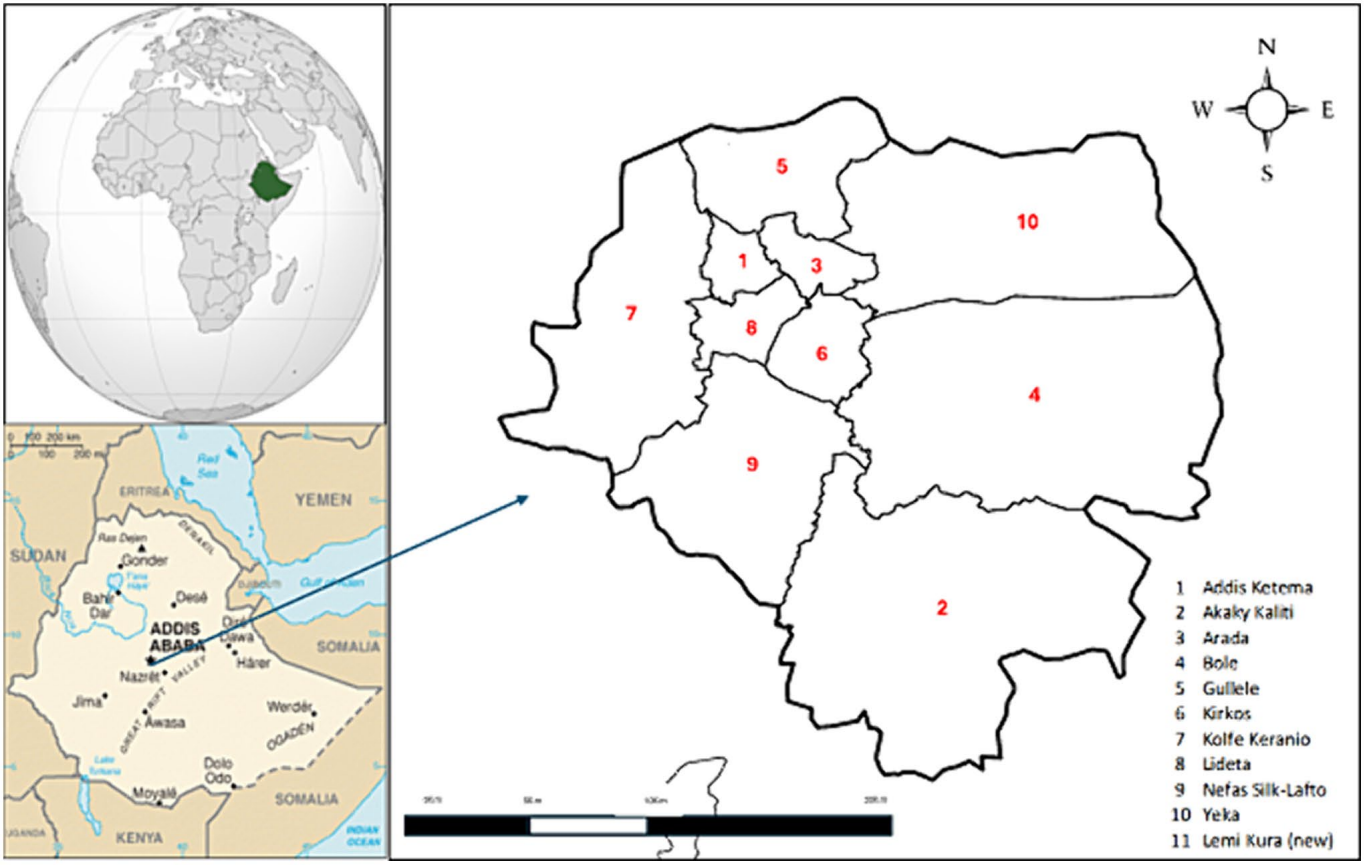
Addis Ababa district locations. This map of the 11 Addis Ababa sub-cities is included in this study. Lemi Kura is a new district and is not included in this map.

This study focuses on Addis Ababa, primarily because of the significant burden of COVID-19 cases. Ethiopia alone has reported 501,157 confirmed cases and 7,574 deaths, with nearly 67% of these cases originating in Addis Ababa (1). Moreover, implementing the COVID-19 HBIC model in Addis Ababa surpasses other regions. Furthermore, the HBIC’s self-reporting and follow-up application is exclusively available in Addis Ababa, making it an ideal location for research. Addis Ababa is divided into 11 sub-cities [Addis Ketema, Akaky Kaliti, Arada, Bole, Gullele, Kirkos, Kolfe Keranio, Lideta, Nefas Silk-Lafto, Yeka, Lemi Kura (New)], all of which have adopted the HBIC system for COVID-19 patients since the initial case was identified. The digital follow-up tool was specifically launched in the Bole sub-city (Figure 1).

### 2.2 Study period

The study was conducted from 1 to 30 April 2022 in Addis Ababa, Ethiopia.

### 2.3 Telemedicine medical team and technology

Each sub-city is equipped with two to four health centers that serve as the base for the telemedicine medical team. A well-established system ensures that patient data is promptly transmitted to follow-up teams, enabling healthcare workers to initiate their follow-up procedures without delay. To support this initiative, the Ethiopian Public Health Institute and Addis Ababa Health Bureau allocated a tablet with complimentary airtime to each sub-city proportionate to the needs of the telemedicine medical team.

### 2.4 Population studied

This study encompasses all individuals diagnosed with COVID-19 who were isolated, received care at home, and met the criteria for inclusion. The study population consisted of confirmed COVID-19 cases admitted to HBIC from its inception, aged between 18 and 60, individuals who underwent regular follow-up assessments while at HBIC, and those whose contact information was documented in the registry.

### 2.5 Patient’s independent variables

Patients were classified based on the following criteria: Gender (male, female), Age (<30, 31–40, 41–50, 51–60 years), city of residence, education level (no education and elementary, secondary and diploma, degree and above), occupation (government employee, private employee, self-employee, not working), symptoms (asymptomatic, symptomatic), type of attention (only telephone call—TC, telephone call with in-person visits - in-person-TC), frequency of calls (≤2 calls, ≥3 calls), and frequency of in-person visits (no visits, ≤2 visits, ≥3 visits).

### 2.6 Study design

**Figure 2:**
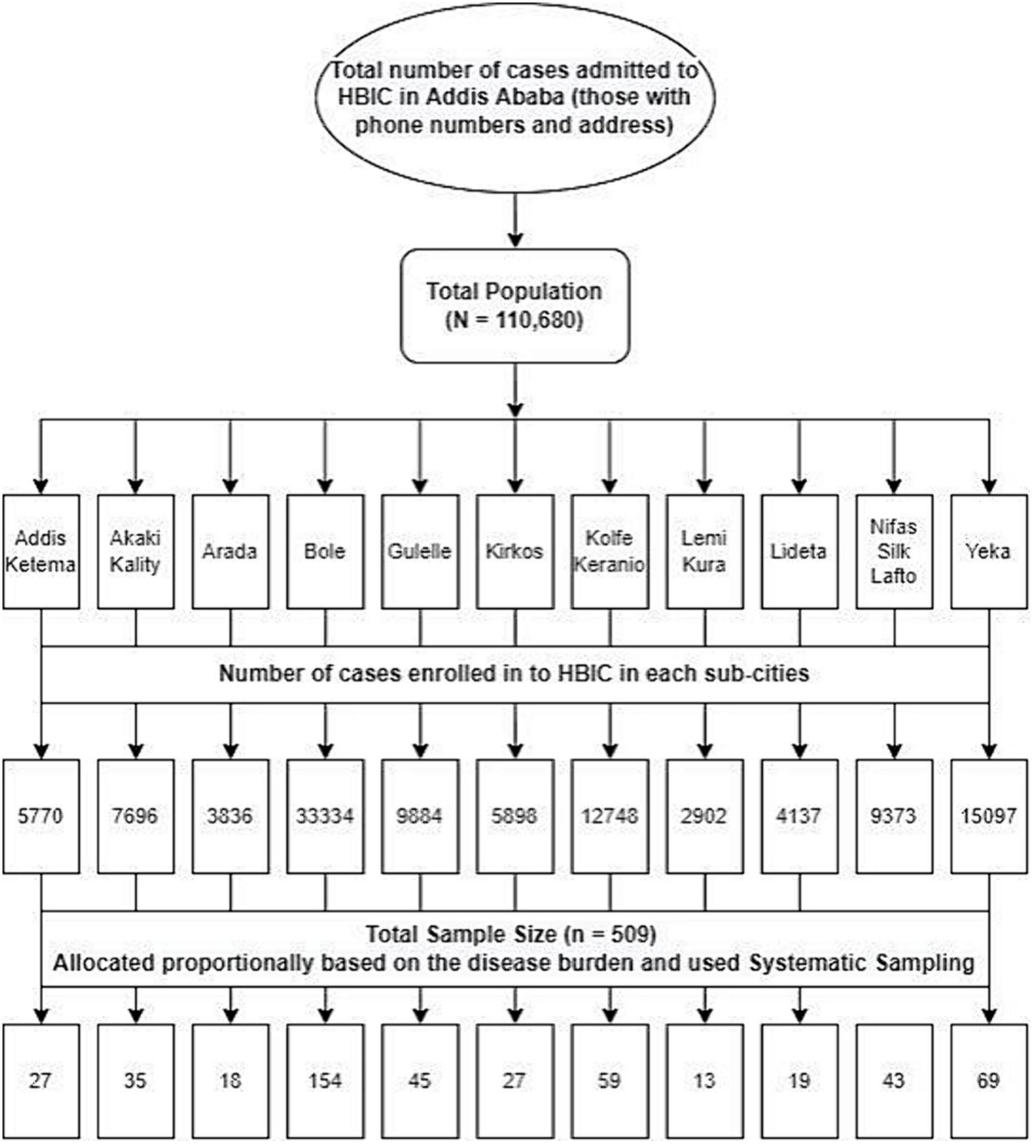
Data collection. Sampling technique from each sub-city in Addis Ababa, Ethiopia.

A cross-sectional research design assessed two primary variables: patient satisfaction and length of stay in HBIC in days. The formula for a single population proportion was applied to determine the required population size of 385 (with a Z-score of 1.96, an outcome proportion of 50%, and a marginal error of 5%). The technique of proportionate stratified sampling involved dividing the entire population into 11 strata or sub-cities. The total sample size was then distributed proportionally among each sub-city based on the burden of the disease. Study participants were selected from each stratum or sub-city using systematic probability sampling (Figure 2).

### 2.7 Data collection tools, techniques, and quality assurance

A validated patient satisfaction survey from the teleneurology program in Chile (13) employed a Likert scale to assess the extent of patient satisfaction. To ensure the accuracy and reliability of the data collected, a pretest was conducted on a random sample of 23 (5%) patients. Based on the findings from the pretest, necessary amendments were made to the data collection tool. Additionally, the collected data underwent daily reviews for clarity and completeness checks. Before analysis, the internal consistency of the questionnaire was evaluated using Cronbach’s alpha test (13). The local language was used during data collection to ensure the required information was collected with greater understanding. To facilitate this, the data collection questionnaire was first developed in English and then translated into Amharic, and the data collected were back-translated to English for consistency. The data collectors administered the questionnaire over the telephone, and responses were recorded on the individual datasheets. Training on the basics of the questionnaire and how to use it appropriately was given by the principal investigator for two BSc nurses for 2 days.

### 2.8 Patient satisfaction survey

The final questionnaire consisted of 17 closed-response questions, each assigned a numerical value ranging from 1 (totally disagree) to 5 (totally agree). The questions were categorized into four contextualized areas. The overall score on the questionnaire, with a maximum of 85 points, served as an indicator of patient satisfaction. The scoring system classified satisfaction levels as follows: very low (17 points or below), low (18–34 points), moderate (35–51 points), high (52–68 points), and very high (69–85 points). Following completion of the survey, patients were divided into two main groups based on their scores: (i) those with a score below 51 points, indicating moderate or low patient satisfaction, and (ii) those with a score over 52 points, indicating high or very high patient satisfaction (Table 1).

**Table 1:** Study questionnaire.

Telemedicine user satisfaction survey
Age:
Gender:
Mark with an × the preferred alternative

		Totally disagree	Disagree	Neutral	Agree	Totally agree
N°	HBIC	1	2	3	4	5
1	I felt comfortable communicating with the follow-up team					
2	Communicating to the follow-up team was as effective as in-person					
3	During the home care, it was easy for me to explain my health problem to the follow-up team					
4	In general, my attention to the system was helpful to me					
5	For the future, I will recommend the system to others					
6	In general, I am satisfied with the care I received					
7	In general, my family is satisfied with the care I received					
8	The system helps me to know my state of health					
9	The system helps me know how to improve my health status					
10	The system allows me to better follow the recommendations and indications of my follow-up team					
11	The follow-up team showed concern in solving my health problem during my stay					
12	The follow-up team was able to answer my questions					
13	The follow-up team has identified my health problem					
14	I have been informed of my right to privacy of my personal and medical information					
15	I prefer this system because it is easier than going to the hospital					
16	I trust that my personal information and privacy will be protected					
17	I trust the instructions of my follow-up team during my stay					

Survey to assess the patient satisfaction treated by COVID-19 home-based isolation and care.

### 2.9 Data and outcomes analysis

Summary statistics, including frequency, percentage, median, and interquartile range, were utilized to summarize the characteristics of patients and other relevant information. This research aimed to assess the relationship between the independent variables and two primary outcomes: (i) the level of patient satisfaction and (ii) the length of stay in the HBIC. The binary logistic regression (Backwald) model was employed to examine the independent variables associated with patient satisfaction, and the findings were presented as odds ratios (OR). Cox regression (Backwald) analysis was conducted to evaluate the independent variables linked to the length of stay in HBIC, and the results were reported as hazard ratios (HR). To compare the means of length of stay in HBIC, a non-normal distribution, the Mann–Whitney U test for independent samples was employed, while the chi-square test was used for categorical variables. The significance level was set at *p* < 0.05. All statistical analyses were performed using SPSS, version 26.

### 2.10 Ethical considerations

The study was conducted after obtaining ethical clearance from the Addis Ababa Health Bureau Public Health Research and Emergency Ethical Review Committee. Oral informed consent was obtained from the participants before any form of data collection. Participants’ contact information was obtained after a formal request was made to the Addis Ababa Health Bureau. Demographic data of all participants and survey responses were anonymously collected and entered. Access to the collected information was limited to the authors, and confidentiality was maintained throughout the project.

## 3 Results

### 3.1 Data collection

**Figure 3:**
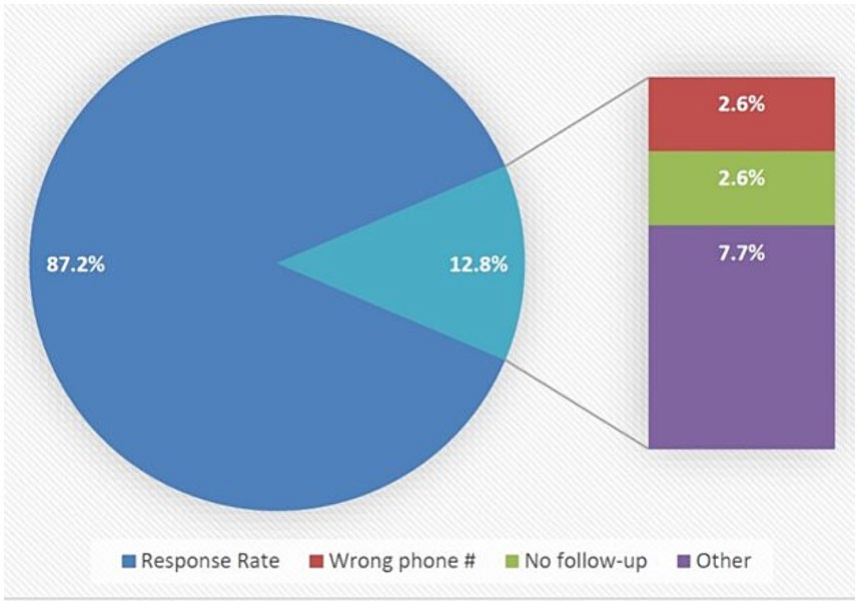
Patient’s response from telephone calls. Response rate and reasons for non-response include: (i) wrong phone number, (ii) no follow-up, and (iii) other.

A 17-question structured questionnaire was developed by reviewing similar studies (Table 1) (13). Upon completing a reliability assessment of the survey, the internal reliability of the survey was robust, as evidenced by a Cronbach’s alpha coefficient of 0.96 (data not shown). The study included 509 patients, yielding a response rate of 87.2% (*n* = 444). Among the non-respondents (*n* = 65, 12.8%), the reasons provided for their lack of participation were as follows: 20% (*n* = 13) had incorrect contact information, and another 20% (*n* = 13) did not receive any follow-up, either in-person or through phone communication. The remaining 60% (*n* = 39) of participants displayed uncooperative behavior, as their phones were inactive, unreachable, or did not answer (Figure 3).

### 3.2 Population description, patient satisfaction, and length of stay in HBIC

The descriptive statistics of the 444 patients who participated in the study are below. The median age was 34 ± 15 years, with an age distribution as follows: Age:<30 (*n* = 164, 36.9%); 31–40 (*n* = 141, 31.8%); 41–50 (*n* = 87, 19.6%); and 51–60 (*n* = 52, 11.7%). The gender distribution of the patients was as follows: Female 247 (55.6%) (Table 2). We outline the performance of the HBIC system according to the patient satisfaction parameter. Due to the low frequency obtained in the lower user satisfaction groups (very low, low, and moderate), we grouped patients into two major groups according to the degree of user satisfaction: low or moderate and high or very high. patients with high or very high user satisfaction represent 313 (70.5%). When we break down this group by gender, we see that women have a slightly higher percentage of high or very high user satisfaction than men, with 177 (56.9%) females. However, no statistically significant differences are observed. Analyzing the distribution of patients with high and very high user satisfaction by age, we observe that more than 50% of patients are concentrated in a population under 40 years old (Table 2). The length of stay for patients with high or very high satisfaction varied depending on certain variables. The average length of stay for females was 15 ± 5 days, while for males, it was 16 ± 5 days, and in terms of age groups, patients showed similar lengths between 14 and 16 days (Table 2).

**Table 2:** Patient’s demographic variables.

		Sat low or moderate		Sat high or very-high		Total	
Demographic variables		n, %	Mean, SD	n, %	Mean, SD	n, %	Mean, SD
Gender	Male	61 (13.7%)	14 (±7)	136 (30.6%)	15 (±5)	197 (44.4%)	15 (±6)
	Female	70 (15.8%)	14 (±6)	177 (39.9%)	16 (±5)	247 (55.6%)	15 (±6)
Age	<30	44 (9.9%)	14 (±6)	120 (27.0%)	15 (±5)	164 (36.9%)	15 (±6)
	31–40	40 (9.0%)	13 (±7)	101 (22.7%)	15 (±5)	141 (31.8%)	15 (±5)
	41–50	34 (7.7%)	14 (±6)	53 (11.9%)	17 (±6)	87 (19.6%)	16 (±6)
	51–60	13 (2.9%)	15 (±6)	39 (8.8%)	16 (±5)	52 (11.7%)	16 (±5)
Address sub-city	Arada	11 (2.5%)	13 (±6)	32 (7.2%)	14 (±4)	43 (9.7%)	14 (±4)
	Gulele	26 (5.9%)	13 (±5)	33 (7.4%)	17 (±4)	59 (13.3%)	15 (±5)
	Lideta	13 (2.9%)	11 (±5)	23 (5.2%)	14 (±4)	36 (8.1%)	13 (±5)
	Yeka	12 (2.7%)	17 (±5)	18 (4.1%)	15 (±7)	30 (6.8%)	16 (±6)
	Akaki Kality	9 (2.0%)	16 (±8)	35 (7.9%)	16 (±8)	44 (9.9%)	16 (±7)
	Nefas Silk Lafto	2 (0.5%)	19 (±15)	20 (4.5%)	20 (±7)	22 (5.0%)	20 (±7)
	Addis Ketema	14 (3.2%)	15 (±9)	48 (10.8%)	17 (±5)	62 (14.0%)	16 (±6)
	Kolfe Keranyo	11 (2.5%)	14 (±6)	33 (7.4%)	16 (±6)	44 (9.9%)	16 (±6)
	Lemi Kura	8 (1.8%)	15 (±7)	8 (1.8%)	14 (±7)	16 (3.6%)	15 (±7)
	Kirkos	5 (1.1%)	14 (±6)	6 (1.4%)	17 (±5)	11 (2.5%)	16 (±6)
	Bole	20 (4.5%)	14 (±7)	57 (12.8%)	14 (±3)	77 (17.3%)	14 (±4)
Education	No educ and elementary	34 (7.7%)	12 (±7)	45 (10.1%)	14 (±7)	79 (17.8%)	13 (±7)
	Secondary and diploma	49 (11.0%)	14 (±6)	125 (28.2%)	15 (±4)	174 (39.2%)	14 (±5)
	Degree and above	48 (10.8%)	16 (±6)	143 (32.2%)	17 (±5)	191 (43.0%)	17 (±6)
Occupation	Government employee	41 (9.2%)	13 (±6)	80 (18.0%)	14 (±5)	121 (27.3%)	14 (±5)
	Private employee	29 (6.5%)	15 (±7)	85 (19.1%)	15 (±5)	114 (25.7%)	15 (±6)
	Self-employee	40 (9.0%)	13 (±6)	80 (18.0%)	16 (±4)	120 (27.0%)	15 (±5)
	Not working	21 (4.7%)	16 (±7)	68 (15.3%)	18 (±7)	89 (20.0%)	18 (±7)
Symptom	Asymptomatic	71 (16.0%)	12 (±5)	98 (22.1%)	14 (±5)	169 (38.1%)	13 (±5)
	Symptomatic	60 (13.5%)	17 (±7)	215 (48.4%)	16 (±5)	275 (61.9%)	16 (±6)
In-person follow-up	No	113 (25.5%)	13 (±6)	217 (48.9%)	15 (±5)	330 (74.3%)	14 (±5)
	Yes	18 (4.1%)	21 (±7)	96 (21.6%)	18 (±6)	114 (25.7%)	19 (±6)
Number of phone calls	<2 calls	92 (20.7%)	12 (±5)	129 (29.1%)	13 (±3)	221 (49.8%)	12 (±4)
	≥3 calls	39 (8.8%)	20 (±6)	184 (41.4%)	18 (±6)	223 (50.2%)	18 (±6)
Number of in-person visits	No visit	113 (25.5%)	13 (±6)	217 (48.9%)	15 (±5)	330 (74.3%)	14 (±5)
	1 visit	14 (3.2%)	19 (±6)	70 (15.8%)	17 (±5)	84 (18.9%)	17 (±5)
	>2 visits	4 (0.9%)	28 (±0)	26 (5.9%)	22 (±7)	30 (6.8%)	23 (±7)

All 444 patients were categorized as follows: patient sex, age, address sub-city, education, occupation, symptom, in-person follow-up, number of calls, and number of in-person visits (n, %). Patients were grouped according to their satisfaction level: Low or moderate and high or very high, frequencies expressed as n, %. Length of stay in HBIC within each group was expressed as mean and standard deviation (SD).

The distribution of patients according to sub-city of residence was found to be quite homogeneous, with some exceptions as detailed below: City: *Addis Ketema (n = 62, 14.0%), Akaki Kality (n = 44, 9.9%), Arada (n = 43, 9.7%), Bole (n = 77, 17.3%), Gulele (n = 59, 13.3%), Kirkos (n = 11, 2.5%), Kolfe Keranyo (n = 44, 9.9%), Lideta (n = 36, 8.1%), Nefas Silk Lafto (n = 22, 5.0%), Yeka (n = 30, 6.8%), and Lemi Kura (n = 16, 3.6%).* Patients with high or very high satisfaction were distributed according to sub-city of residence as follows: City: *Addis Ketema (n = 48, 10.8%), Akaki Kality (n = 35), Arada (n = 32, 7.2%), Bole (n = 57, 12.8%), Gulele (n = 33, 7.4%, 7.9%), Kirkos (n = 6, 1.4%), Kolfe Keranyo (n = 33, 7.4%), Lideta (n = 23, 5.2%), Nefas Silk Lafto (n = 20, 4.5%), Yeka (n = 18, 4.1%), and Lemi Kura (n = 8, 1.8%)* (Table 2). Regarding the city of origin, the average length of stay varied as follows: *Addis Ketema (17 ± 5), Akaki Kality (16 ± 8), Arada (16 ± 5), Bole (14 ± 3), Gulele (17 ± 4), Kirkos (17 ± 5), Kolfe Keranyo (16 ± 6), Lideta (14 ± 4), Nefas Silk Lafto (20 ± 7), Yeka (15 ± 7), and Lemi Kura (14 ± 7)* (Table 2).

The patient’s satisfaction and length of stay in HBIC can be influenced by various factors, including the patient’s level of education and occupation. The distribution of patients’ education level is as follows: 79 patients (17.8%) had no education or only completed elementary school, 174 patients (39.2%) had completed secondary school or obtained a diploma, and 191 patients (43.0%) had a degree or higher education. The distribution of patients’ occupation is as follows: 121 patients (27.3%) were government employees, 114 patients (25.7%) were private employees, 120 patients (25.0%) were self-employed, and 89 patients (20.0%) were not working (Table 2). The majority of patients who showed high or very high user satisfaction have higher levels of education: Education: No Educ and Elementary (*n* = 45, 10.1%), Secondary and Diploma (*n* = 125, 28.2%), Degree and above (*n* = 143, 32.2%); however, the distribution was found to be more symmetrical according to the occupational level of these patients: Government employee (*n* = 80, 18.0%), Private employee (*n* = 85, 19.1%), Self-employee (*n* = 80, 18.1%), Not working (*n* = 68, 15.3%) (Table 2). No significant differences were found in the duration length of stay in HBIC among patients concerning their level of education and occupation type. The respective data for education are as follows: No Education and Elementary 14 ± 7, Secondary and Diploma 15 ± 4, Degree and above 17 ± 5. Similarly, the data for occupation type were: Government employee 14 ± 5, Private employee 15 ± 5, Self-employee 16 ± 4, Not working 18 ± 7 (Table 2).

Most patients who participated in this study were symptomatic, accounting for 275 (61.9%) with an average length of stay for HBIC of 16 (±6). Patients who displayed symptoms were more satisfied (*n* = 215, 48.5%) than those who were asymptomatic (*n* = 98, 22.1%). The length of stay in HBIC for asymptomatic patients (14 ± 5), was slightly lower than that of Symptomatic patients (16 ± 5). However, no statistically significant differences were observed (data not shown).

Next, we described the frequency of phone call follow-ups for patients during their stay at HBIC. The most common type of care was TC, with 330 patients (74.3%), followed by in-person and TC visits, with 114 patients (25.7%). The overall frequency of in-person visits was 1 ± 2 visits, and it can be further broken down as follows: no visits (*n* = 330, 48.9%), ≤2 visits (*n* = 84, 18.9%), and ≥ 3 visits (*n* = 30, 6.8%). The average frequency of phone call follow-ups was 3 ± 6 calls, and we observe that the distribution of calls appears to be symmetrical: TC was ≤2 calls (*n* = 221, 49.8%) and ≥ 3 calls (*n* = 223, 50.2%).

In terms of the mode of care, patients who were exclusively contacted through teleconsultation (TC) 217 (48.9%) were more satisfied than those receiving hybrid care with both TC and in-person visits 96 (21.6%). Consistent with the findings, patients exhibited higher user satisfaction with an increased frequency of calls in TC, as those patients with more than ≥3 calls (*n* = 184, 41.4%) expressed greater satisfaction compared to patients with less than or equal to ≤2 calls (*n* = 129, 29.1%) (Table 2). Upon analyzing the frequency of in-person visits, patients with fewer visits reported higher user satisfaction: No visits (*n* = 217, 69.3%), 1 visit (*n* = 70, 22.0%), and ≥ 2 visits (*n* = 26, 8.3%).

It is important to mention that differences in patient length of stay were observed according to the type of care, as patients treated through the TC system 14 ± 5 stayed for a shorter period than those receiving hybrid care through TC and in-person visits 18 ± 6 (*p* < 0.05). As expected, patients with more calls stayed longer in the HBIC system as described below: Frequency of calls in TC: ≤2 calls 13 ± 3, and ≥ 3 calls 18 ± 6 (*p* < 0.05) (Table 2).

### 3.3 Statistical analysis associated with patient satisfaction

A binary logistic regression analysis was utilized to explore the variables associated with high or very high patient satisfaction levels. The results revealed that individuals who placed more than three calls (OR = 2.827, 95% CI = 1.798, 4.443, *p* = 0.000) and those displaying symptoms (OR = 2.001, 95% CI = 1.289, 3.106, *p* = 0.002) were more inclined to report increased satisfaction. Nevertheless, no statistically significant relationships were found between user satisfaction and the other independent variables investigated (*p* > 0.05) (Table 3).

**Table 3:** Relevant demographic variables that influence patient satisfaction.

Binary logistic regression (Backwald)					
	B	Sig.	Exp(B)	95% IC. for EXP(B)	
				Lower	Upper
Symptomatic	0.694	**0.002**	2.001	1.289	3.106
Number of phone calls (≥3 calls)	1.039	**0.000**	2.827	1.798	4.443

Binary logistic regression (Backwald) variables from step 1 were: sex of the patient, age, address sub-city, education, occupation, symptom, in-person follow-up, number of calls, and number of in-person visits. The variables symptom (OR = 2.001) and number of calls (≥3 calls) (OR = 2.827) were shown to be statistically significant in the model (*p* < 0.05). Bold numbers are the *p* values.

### 3.4 Statistical analysis associated with patient length of stay in HBIC

A Cox regression analysis was employed to examine the factors influencing the duration of hospital stays in patients who reported high levels of satisfaction. The findings revealed that patients who made more than three phone calls had a significantly lower hazard ratio (HR = 0.537, 95% CI = 0.415, 0.696, *p* = 0.000), indicating a greater likelihood of reducing their length of stay. Similarly, patients with more in-person visits also exhibited a lower hazard ratio (HR = 0.495, 95% CI = 0.322, 0.762, *p* = 0.001). However, no statistically significant associations were observed between the length of stay and the other independent variables analyzed (*p* > 0.05) (Table 4).

**Table 4:** Cox regression analysis to elucidate the demographic variables associated with the length of stay in HBIC and patients’ satisfaction.

Length of stay in HBIC analysis with Cox regression					
	B	Sig.	Exp(B)	95,0% CI for Exp(B)	
				Lower	Upper
Number of phone calls	−0.621	0.000	0.537	0.415	0.696
Number of in-person visits (0)		0.006			
Number of in-person visits (1 visit)	−0.076	0.596	0.927	0.699	1.228
Number of in-person visits (≥2 visits)	−0.703	0.001	0.495	0.322	0.762

The variables analyzed from step 1 were: sex of the patient, age, address sub city, education, occupation, symptom, in-person follow-up, number of calls, and number of in-person visits. The variables number of calls (HR = 0.537) and number of in-person visits (≥3 calls) (HR = 0.495) were shown to be statistically significantly associated with a reduction in the Length of stay in HBIC (*p* < 0.05).

### 3.5 Statistical analysis of the variables affecting patient satisfaction and length of stay in HBIC

Previously, we identified the following variables that significantly influence patient satisfaction and length of stay in HBIC: the presence of symptoms, the number of phone calls, and in-person follow-ups. Subsequently, we examined the relationship between symptoms and patient satisfaction with the number of phone calls and in-person follow-ups (Table 5). It was noted that symptomatic patients with low or moderate satisfaction levels have an OR = 5.7 times higher likelihood of having a greater number of phone calls and 7.6 times higher likelihood of having in-person follow-ups. The length of stay in HBIC for these patients was notably longer for those with a higher number of phone calls. Conversely, patients with high or very high satisfaction levels have a 2.3 times higher likelihood of having a greater number of phone calls and in-person follow-ups. The length of stay in HBIC for these patients was significantly longer for those with in-person follow-up. Sig *p* < 0.05 (Table 5).

**Table 5:** Analysis of the variables affecting patient satisfaction and length of stay in HBIC.

			Asymptomatic		Symptomatic				
			n,%	Length of stay in HBIC	n,%	Length of stay in HBIC	OR	p frequency	p length of stay in HBIC
Sat low or moderate									
	Number of phone calls	<2 calls	61 (13.7%)	10 (±3)	31 (7%)	14 (±6)	5.7	0.00	0.00
		≥3 calls	10 (13.7%)	21 (±5)	29 (7%)	20 (±7)			
	In-person follow-up	No	68 (13.7%)	11 (±5)	45 (7%)	16 (±6)	7.6	0.00	0.00
		Yes	3 (13.7%)	19 (±8)	15 (7%)	21 (±7)			
Sat high or very high									
	Number of phone calls	<2 calls	54 (13.7%)	12 (±3)	75 (7%)	13 (±3)	2.3	0.00	
		≥3 calls	44 (13.7%)	17 (±5)	140 (7%)	18 (±6)			
	In-person follow-up	No	79 (13.7%)	14 (±5)	138 (7%)	15 (±4)	2.3	0.00	
		Yes	19 (13.7%)	16 (±2)	77 (7%)	19 (±6)			0.00

The study examined the relationship of variables that significantly affected the patient satisfaction and length of stay in HBIC. It was observed that symptomatic patients with Low or Moderate satisfaction levels have an OR = 5.7 times higher chance of having a greater Number of Phone Calls and 7.6 times higher chance of having In-Person follow-up. The length of stay in HBIC for these patients was significantly longer for those with a higher number of phone calls. On the other hand, patients with high or very high satisfaction levels have a 2.3 times higher chance of having a greater number of phone calls and in-person follow-up. The length of stay in HBIC for these patients was significantly longer for those with in-person follow-up. Sig *p* < 0.05.

## 4 Discussion

This study aimed to evaluate the levels of patient satisfaction and length of stay in HBIC among COVID-19 patients while also examining the different factors that may impact these outcomes. Given the considerable number of COVID-19-positive instances and the limited bed availability at COVID-19-specific hospitals, the HBIC endorsed the practice of home isolation for COVID-19-affected persons. This directive was instituted because most COVID-19 patients were either asymptomatic or displayed mild symptoms. Such cases generally do not necessitate hospitalization at COVID-19-designated medical facilities and can be adequately managed at home with appropriate medical guidance and monitoring. The heightened level of patient satisfaction observed in symptomatic individuals treated by HBIC is supported by an associated investigation conducted through a survey-based study (10, 16–18).

We identified a noteworthy connection between a high or very high satisfaction level and the frequency of phone call follow-ups in patients with COVID-19 symptoms. Essentially, for each increment of one in the frequency of phone call follow-ups, the chances of experiencing high or very high patient satisfaction rose by a factor of 2.8 (OR = 2.827, 95% CI = 1.798, 4.443, *p* = 0.000). This tendency may be attributed to patients encountering difficulties during their healthcare facility stay, and regular communication with the follow-up team assists in addressing some of these challenges. As per the National HBIC Guideline, the frequency of phone call follow-ups escalates as the severity of the COVID-19 illness worsens, with follow-ups occurring once a week, every 3 days until discharge, and daily until discharge (19). Correspondingly, individuals exhibiting COVID-19 symptoms had 2.0 times greater odds of experiencing high or very high patient satisfaction than those with asymptomatic disease (OR = 2.001, 95% CI = 1.289, 3.106, *p* = 0.002). Cox regression analysis uncovered a negative correlation between the length of stay in HBIC patients with high or very high satisfaction and the frequency of phone calls, follow-ups, and in-person visits. Specifically, the number of phone calls and in-person visits (with a minimum of two visits) emerged as significant factors impacting the length of stay. Upon adjusting for other variables, it was observed that with each additional phone call, the length of stay in the hospital-based isolation center (HBIC) decreased by 46.3% (HR = 0.537, 95% CI = 0.415, 0.696, *p* = 0.000). Similarly, after accounting for other covariates, the length of stay among COVID-19 patients who had more than one in-person visit was 50.5% lower compared to patients with no in-person visits (HR = 0.495, 95% CI = 0.322, 0.762, *p* = 0.001). Finally, this investigation determined that symptomatic patients with Low or Moderate satisfaction had a greater likelihood of experiencing a larger Number of Phone Calls, In-Person follow-ups, and a longer stay at HBIC compared to patients with high or very high satisfaction.

User satisfaction and length of stay have shown dissimilar results in different latitudes. A study conducted in general and university hospitals in the Netherlands found no evidence of a correlation between the average length of stay in hospital wards and patient satisfaction (20). Several studies conducted at the Nancy University Hospital Center in France and in training hospitals in Turkey and Japan (21–23) have found a connection between prolonged lengths of stay and decreased patient satisfaction. These studies have highlighted the importance of environmental factors in influencing patient satisfaction (24). However, a distinct study carried out in teaching hospitals in Turkey discovered that patients who stayed longer expressed higher satisfaction levels than those with shorter stays (25). Despite these variations, one can speculate about the reasons for higher user satisfaction in patients with greater follow-ups. Among these reasons, it can be argued that it is well-documented that telemedicine technology has the potential to enhance the quality of primary medical care, shorten the duration of treatment, and decrease the number of severe hospitalization cases (17). Telemedicine expands the availability of medical services and broadens the range of medical care, which is particularly crucial in Ethiopia, with a fragmented healthcare system and limited coverage (26, 27). The sharing of medical resources is especially significant for rural or isolated areas, and telemedicine offers a greater abundance of advanced medical resources for primary hospital consultations in these vast rural regions, thereby enhancing the quality of medical and healthcare services (10, 28, 29).

The incorporation of telemedicine into the healthcare systems of low-and middle-income countries (LMICs), such as Ethiopia, has the potential to bring about cost savings and resource conservation in the long run. Consequently, this could alleviate the financial burden on individuals and enhance their access to affordable healthcare services (17). It is important to note that in many LMICs, a significant portion of overall health spending comes from out-of-pocket payments, as there is often no general health insurance available (30). Moreover, the COVID-19 pandemic has further strained the already fragile healthcare systems in LMICs (10, 31). In this regard, telemedicine services could be crucial in relieving pressure on the healthcare system by saving time and resources and enhancing efficiency and accessibility. Additionally, telemedicine can facilitate social distancing measures and reduce the need for face-to-face interactions in hospitals and clinics, helping prevent the spread of infectious diseases like COVID-19 through physical contact (10, 32). Furthermore, telemedicine can also be instrumental in providing counseling and specific advice to patients during the COVID-19 pandemic, such as guidance on prevention measures. Overall, the adoption of telemedicine in LMICs holds great potential for addressing healthcare challenges, reducing costs, and improving access to quality care. It can serve as a valuable tool in mitigating the impact of the COVID-19 pandemic and strengthening healthcare systems in these countries (33).

Throughout the initial three waves of the COVID-19 pandemic in Ethiopia, most patients seeking medical care were directed to the HBIC. This strategic decision is believed to have effectively alleviated the potential burden of cases and prevented burnout among the existing treatment facilities (2). The HBIC specifically caters to COVID-19 patients and represents our country’s pioneering national telehealth service. Therefore, it is crucial to examine its operational experiences thoroughly. The focus should be on integrating functionalities that encourage community acceptance of this telehealth modality. As COVID-19 patients continue to be enrolled in the HBIC, the findings from this study will serve as a valuable resource for the Ministry of Health and COVID-19 response teams in their efforts to enhance the healthcare system. The HBIC framework offers a practical solution for healthcare providers who aim to extend their services to patients residing in remote areas. Moreover, the growing acceptance and familiarity with telemedicine visits, driven by the pandemic, are likely to shape the future landscape of healthcare delivery for both patients and providers.

The study’s findings should be evaluated considering the strengths and limitations identified. The study’s strength lies in the thorough selection process of participants from various sub-cities throughout the entire duration of the service. The research questions were also carefully developed using a validated tool and assessed for internal consistency, ensuring their comprehensibility.

### 4.1 Limitations

It is important to acknowledge a limitation of the study, which is the potential recall bias introduced by enrolling most patients during the initial phase of the COVID-19 pandemic. Despite this limitation, this study is expected to pave the way for advancements in telemedical services in Ethiopia. Furthermore, it can potentially expand and incorporate other telemedicine care modalities, such as videoconferencing, in the future.

## 5 Conclusion

The investigation evaluated patient satisfaction and length of stay in telemedicine services. The findings revealed that most patients, accounting for over 70.5%, reported high or very high satisfaction with these services. Notably, patients who received frequent follow-up phone calls and exhibited symptomatic COVID-19 disease expressed higher satisfaction. Additionally, it was observed that a higher frequency of phone calls and in-person visits resulted in a shorter stay in the Hospital-Based Isolation Center (HBIC). Consequently, it is crucial to consider the aforementioned factors to enhance patient satisfaction and reduce the length of stay in HBIC or other telemedicine services. It is worth mentioning that the HBIC, which caters specifically to COVID-19 patients, represents the first large-scale national telemedicine service implemented in our country. Therefore, it is imperative to thoroughly examine the experiences gained from this service, with particular attention given to incorporating features that facilitate community acceptance and reimbursement of this mode of healthcare delivery. Meanwhile, as the enrollment of COVID-19 patients in the HBIC continues, the findings of this study will serve as a valuable resource for guiding the Ministry of Health, the Ethiopian Public Health Institute, and the COVID-19 response team in their efforts to improve the system.

## Data availability statement

The original contributions presented in the study are included in the article/Supplementary material, further inquiries can be directed to the corresponding authors.

## Ethics statement

The studies involving humans were approved by the Addis Ababa Health Bureau Public Health Research and Emergency Ethical Review Committee. The studies were conducted in accordance with the local legislation and institutional requirements. The participants provided their written informed consent to participate in this study.

## Author contributions

DG: Writing – review & editing, Writing – original draft, Visualization, Validation, Supervision, Software, Resources, Project administration, Methodology, Investigation, Formal analysis, Data curation, Conceptualization. ZA: Writing – review & editing, Writing – original draft, Visualization, Validation, Supervision, Software, Resources, Project administration, Methodology, Investigation, Formal analysis, Data curation, Conceptualization. FC: Writing – review & editing, Writing – original draft, Visualization, Validation, Supervision, Software, Resources, Project administration, Methodology, Investigation, Formal analysis, Data curation, Conceptualization. FB: Writing – review & editing, Writing – original draft, Methodology, Investigation, Formal analysis, Data curation, Conceptualization. YT: Writing – review & editing, Writing – original draft, Resources, Project administration, Methodology, Investigation, Formal analysis, Data curation, Conceptualization. CM: Writing – review & editing, Writing – original draft, Supervision, Resources, Methodology, Investigation, Formal analysis, Data curation. JR: Writing – review & editing, Writing – original draft, Validation, Project administration, Methodology, Investigation, Formal analysis, Data curation. CA-L: Writing – review & editing, Writing – original draft, Visualization, Validation, Supervision, Software, Resources, Project administration, Methodology, Investigation, Formal analysis, Data curation, Conceptualization.
